# The WHO 2018 Classification of Cutaneous Melanocytic Neoplasms: Suggestions From Routine Practice

**DOI:** 10.3389/fonc.2021.675296

**Published:** 2021-07-02

**Authors:** Gerardo Ferrara, Giuseppe Argenziano

**Affiliations:** ^1^ Anatomic Pathology Unit, Macerata General Hospital, Macerata, Italy; ^2^ Department of Dermatology, ‘Luigi Vanvitelli’ University School of Medicine, Naples, Italy

**Keywords:** melanoma, melanocytoma, dysplastic nevus, clinicopathological correlation, histopathology, immunohistochemistry, molecular biology

## Abstract

The “multidimensional” World Health Organization (WHO) classification 2018 of melanocytic tumors encompasses nine melanoma pathways (seven of which for cutaneous melanoma) according to a progression model in which morphologically intermediate melanocytic tumors are cosidered as simulators and/or precursors to melanoma. These “intermediates” can be subclassified into: i) a “classical” subgroup (superficial/thin compound: dysplastic nevus), which is placed within the morphologic and molecular progression spectrum of classical (Clark’s and McGovern’s) melanoma subtypes (superficial spreading and, possibly, nodular); and ii) a “non-classical” subgroup (thick compound/dermal: “melanocytomas”) whose genetic pathways diverge from classical melanoma subtypes. Such a progression model is aimed at giving a conceptual framework for a histopathological classification; however, routine clinicopathological practice strongly suggests that most melanomas arise *de novo* and that the vast majority of nevi are clinically stable or even involuting over time. Clinicopathological correlation can help identify some severely atypical but benign tumors (*e.g.*: sclerosing nevus with pseudomelanomatous features) as well as some deceptively bland melanomas (*e.g.*: lentiginous melanoma; nested melanoma), thereby addressing some ambiguous cases to a correct clinical management. The recently available adjuvant therapy regimens for melanoma raise the problem of a careful distinction between severely atypical (high grade) melanocytoma and “classical” melanoma: conventional morphology can guide an algorithmic approach based on an antibody panel (anti-mutated BRAF, BAP1, PRAME, ALK, TRKA, MET, HRAS-WT, ROS; beta catenin; R1alpha; p16; HMB45; Ki67), a first-line molecular study (identification of hot spot mutations of *BRAF* and *NRAS*) and an advanced molecular study (sequencing of *NF1, KIT, BRAF, MAP2K1, GNAQ, GNA11, PLCB4, CYSLTR2, HRAS*; fusions studies of *BRAF, RET, MAP3K8, PRKCA*); as a final step, next-generation sequencing can identify melanocytic tumors with rare genetic signatures and melanocytic tumors with a high tumor mutation burden which should be definitely ascribed to the category of classical melanoma with the respective therapeutic options.

## Introduction

The histopathological diagnosis and classification of melanocytic skin tumors is probably the greatest conceptual and practical challenge in modern dermatopathology and is expected to rapidly evolve in the next future, with the WHO 2018 classification being the basis for the forthcoming studies (1). One major problem, however, is that the histopathological diagnosis itself is not based upon the search of a single (or a few), objective, and easily reproducible morphological diagnostic feature(s) but rather, it is born by a constellation of diagnostic criteria whose implementation, meaning, and relative weight considerably vary case by case and is responsible for a worrisome list of diagnostic pitfalls (**Table 1**). Thus, the histopathological diagnosis of melanocytic skin neoplasms, being based upon the simultaneous evaluation of several criteria, is no more than an *assessment of probability* and, as such, is often a matter of a sizable disagreement and inter-observer variability (2). In addition, and even more importantly, the time-honored “unifying concept of melanoma” (melanoma as a single entity evolving with a well-defined and repetitive “sequence of events”) (3) has been questioned, because both clinicopathological (4) and molecular studies (5) point toward the existence of melanocytic neoplasms of low malignant potential (putative low-grade melanocytic malignancies different from “classical” melanoma).

**Table 1:** Main settings of diagnostic difficulties in melanocytic skin neoplasms.

1. Unrecognized melanoma on partial (shave/punch) biopsies
2. Nevoid melanoma *vs*. “common” or “congenital” compound/dermal nevus
3. Desmoplastic melanoma *vs*. desmoplastic nevus *vs*. scar
4. Recurrent/persistent nevus *vs*. (recurrent) melanoma
5. Spindle cell melanoma *vs*. spindle cell nevus
6. Spitz/spitzoid melanoma *vs*. atypical Sptz nevus/tumor *vs*. Spitz nevus
7. Superficial spreading melanoma *vs*. dysplastic nevus
8. Superficial spreading melanoma *vs*. haloed nevus
9. Melanoma (in special site) *vs*. nevus with site-related atypia
10. Melanoma with regression *vs*. compound nevus with regression-like fibrosis
11. Melanoma with regression *vs*. melanosis
12. Melanoma *in situ* in chronic sun-damaged skin *vs*. melanocytic hyperplasia/photoactivation
13. Dermal melanoma over congenital nevus *vs*. proliferative nodule in congenital nevus
14. Cellular blue nevus *vs*. animal-type melanoma *vs*. blue nevus-like metastatic melanoma
15. Deep penetrating nevus *vs.* deep penetrating nevus-like melanoma
16. Pigmented epithelioid melanocytoma *vs*. animal-type melanoma

In order to face with these problems in routine histopathological practice, the WHO Working Group supports the use of descriptive and provisional terminology, *i.e:* i) “intraepidermal atypical melanocytic proliferation of uncertain significance (IAMPUS)”: a melanocytic neoplasms raising the differential diagnosis with melanoma *in situ*; ii) “superficial atypical melanocytic proliferation of uncertain significance (SAMPUS)”: a thin compound melanocytic neoplasm whose differential diagnosis is with early invasive, radial growth phase (thin non-mitogenic and non-tumorigenic) melanoma; iii) “melanocytic tumor of uncertain malignant potential (MELTUMP)”: a compound or dermal-based neoplasm whose differential diagnosis includes melanoma in vertical growth phase (typified by dermal mitotic figures and/or by dermal nests/sheets which are larger than the larger junctional nest) (6). Based on the these definitions, such a descriptive terminology applies to simulators (morphologically atypical nevi and deceptively bland melanomas) (2) as well as to biological “intermediates” (melanocytic neoplasms of low malignant potential) (4); and a strong suggestion is made that several neoplasms belonging to both categories may be in fact precursors to melanoma. The present review is aimed at giving some suggestions in the multidisciplinary approach based on the WHO 2018 classification.

## The Pathways to Melanoma

The WHO 2018 classification of melanocytic tumors sets forth nine pathways to melanoma (6), seven of which being primary cutaneous (**Table 2**), by largely transposing a previously proposed “multidimensional” pathogenetic scheme based on: i) the role of ultraviolet (UV) radiation; ii) the cell (or tissue) of origin; iii) driving and/or recurrent genomic changes (7).

**Table 2:** The WHO 2018 classification of melanoma according to pathways.

Relationship with sun exposure/sun damage	Pathway n.	Subtype	Genetic hallmarks
Melanomas arising in sun-exposed skin	1	*Low-CSD melanoma/superficial spreading melanoma*	High frequency of *BRAF* p.V600 mutations (7–9)
	2	*High-CSD melanoma (including lentigo maligna melanoma and high-CSD nodular melanoma)*	Predominating mutually exclusive *NF1*, *NRAS*, other *BRAF* (non-p.V600E), and perhaps *KIT* mutations (7–9)
	3	*Desmoplastic melanoma*	Recurrent inactivating *NF1* mutations, *NFKBIE* promoter mutations, and several different activating mutations in the MAPK pathway (*e.g*.: *MAP2K1*) (9–11)
Melanomas arising at sun-shielded sites or without known etiological associations with UV radiation exposure	4	*Malignant Spitz tumor (Spitz melanoma)*	Mutations in *HRAS* and kinase fusions in *ROS1*, *NTRK1*, *NTRK3*, *ALK*, *BRAF*, *MET*, and *RET*; *CDKN2A* homozygous deletion, *TERT* promoter mutations and *MAP3K8* fusions/truncating mutations only in aggressive or lethal variants (7, 12–15)
	5	*Acral melanoma (including nodular melanoma in acral skin)*	Multiple amplifications of *CCND1*, *KIT*, and *TERT*; mutations of *BRAF*, *NRAS*, and *KIT*; kinase fusions of *ALK* or *RET* in a few cases (7, 8)
	6	*Mucosal melanoma*	Numerous copy number and structural variations; uncommonly, *KIT* and *NRAS* mutations (16)
	7	*Melanoma arising in congenital nevus*	In large to giant congenital nevi: *NRAS* mutation; in small to medium-sized congenital nevi, *BRAF* mutations (17, 18)
	8	*Melanoma arising in blue nevus*	Initiating mutations in the Gαq signalling pathway (*GNAQ*, *GNA11, CYSLTR2, PLCB4*); monosomy 3 (associated with loss of *BAP1*) and chromosome 8q gains in aggressive cases; additional secondary copy number aberrations in *SF3B1* and *EIF1AX* (7, 19)
	9	*Uveal melanoma*	Mutually exclusive mutations in the G*α*q pathway (*GNAQ*, *GNA11*, *PLCB4*, *CYSLTR2*); *BAP1*, *SF3B1*, and *EIF1AX* mutations during progression (16)

The most common melanomas in Whites arise from epithelium-associated melanocytes in cutaneous sites with some degree of cumulative sun damage (CSD); these neoplasms are characterized by a high number of point mutations, mostly consisting in the so-called “UV signature” (cytosine to thymidine transitions at dipyrimidine sites); as a rule, the higher the degree of CSD the higher the tumor mutation burden (TMB) (on average: 30 mutations/megabase in high-CSD melanoma; 15 mutations/megabase in low-CSD melanoma) (10). Desmoplastic melanoma is a subtype of high-CSD characterized by a particularly high TMB (on average: 62 mutations/megabase) (11). The degree of CSD is related with the histopathological evidence of dermal solar elastosis, graded according to a three-tiered scale (grade 1: single elastic fibers; grade 2: bunches of fibers; grade 3 basophilic masses) (6).

The other subtypes of melanoma are UV-unrelated. The most common melanomas in non-White population arise from epithelium-asssociated melanocytes on acral skin (palms, soles, nail apparatus) or mucous membranes and are characterized by an early onset of major chromoscomal rearrangements, such as chromotripsis, with gene copy number changes, including multiple high-level amplifications (8). Spitz melanoma and melanomas arising from non-epithelium associated melanocytes (uveal melanoma, melanoma arising in blue nevus and in congenital nevus) also have a very low TMB, but lack the highly rearranged genomes of acral and mucosal melanomas (7, 20). The separation among melanomas with different TMBs is clinically relevant because the TMB may be predictive of response to immune checkpoint inhibitors (21, 22); parenthetically, the assessment of the TMB may be even proposed as a tool for the management of some cases of severely atypical MELTUMP (see below).

Next generation sequencing (NGS) studies have identified many recurrently mutated genes in melanoma, incuding well known genes (*PTEN*, *MAP2K1-2*, *RB1*) and recently identified genes (*ARID2*, *PPP6C*, *RAC1*, *DDX3X*, *IDH1*) (23, 24); however, most of these genes are involved in melanoma progression, rather than in melanoma initiation. Based on the presence of specific driver mutations, The Cancer Genome Atlas (TCGA) classified melanomas into four molecular subtypes: *BRAF*-mutated, *RAS*-mutated, *NF1*-mutated, and triple wild-type (lack of mutations in all three genes); among the latter were cases characterized by *KIT* mutations and by early onset of somatic copy number variations in terms of both gene amplifications in *KIT*, *CCND1*, *CDK4*, *MITF*, and *TERT* and gene deletion/loss-of-function of *TP53* and *CDKN2A* (9).

TCGA molecular subtypes correspond to most cases of the classical (Clark’s and McGovern’s) (25, 26) types of melanoma and roughly identify melanoma pathways 1–3 of the WHO 2018 classification; melanoma arising in congenital nevus may be also genetically related to classical melanoma because they harbor multiple DNA copy number changes (17) superimposed to *NRAS* mutation. By contrast, the genetic profiles of Spitz melanoma (mutations in *HRAS* and kinase fusions in *ROS1*, *NTRK1*, *NTRK3*, *ALK*, *BRAF*, *MET*, and *RET*) (12, 13) as well as of melanoma arising in blue nevus (mutations in the G*α*q signalling pathway) (19, 27) are not encompassed within the TCGA classification. Such cases will unlikely harbor numerous DNA copy number changes or a high TMB; thus they may be genetically considered as “non-classical” subtypes of melanoma.

## Nevi as Potential Precursors to Melanoma

As a rule, all nevi may be virtually simulators of melanoma (and *vice versa*). In addition, the recent identification of the presence of shared genomic abnormalities between some melanomas and associated nevi has provided support for a potential role of some nevi (28) as both simulators and precursors. However, only some of the WHO 2018 pathways to melanoma may have their putative startpoint in nevi harboring the same mutation:

- Pathway 1: the vast majority of acquired nevi possess single driver mutations of either *BRAF* V600E or *NRAS* Q61R/L (29);
- Pathway 4: some Spitz nevi harbor *HRAS* mutation or translocations with kinase gene fusions involving *ALK*, *ROS*, *RET*, *MET*, and *NTRK* (12, 13).
- Pathway 7: *NRAS* mutation is most frequently observed in congenital melanocytic nevi (18);
- Pathway 8: some blue nevi harbor the *GNAQ* or *GNA11* mutation (19, 27).

In contrast to melanomas, which acquire additional driver mutations, nevi usually enter a suppressive state of replicative senescence which is regulated by the tumor suppressor gene *CDKN2A via* its proteins, p14 and p16, and various transcriptional controls of the cell cycle (30, 31). Therefore, the above-listed mutations, as a single event, appear to be insufficient for melanomagenesis, but bear partially transformed melanocytes which may have an increased susceptibility to additional pathogenic mutation(s) (16). Such a progression model also encompasses neoplasms that have an intermediate number of pathogenetic mutations between nevi and melanomas: within this category, the WHO Working Group lists atypical junctional/thin compound neoplasms (dysplastic nevus and melanoma *in situ*) as well as papulonodular tumorigenic dermal proliferations (“melanocytomas”), and both categories are subclassified into low-grade and high-grade (16). Like Pathway 1 to melanoma, dysplastic nevi are associated with activating mutations of *BRAF* or *NRAS* (18, 29); additional mutation of the *TERT* promoter and, sometimes, hemizygous loss of *CDKN2A* are involved in the morphological progression to a “classical” (superficial spreading) melanoma *in situ* (32).

**Figure 1:**
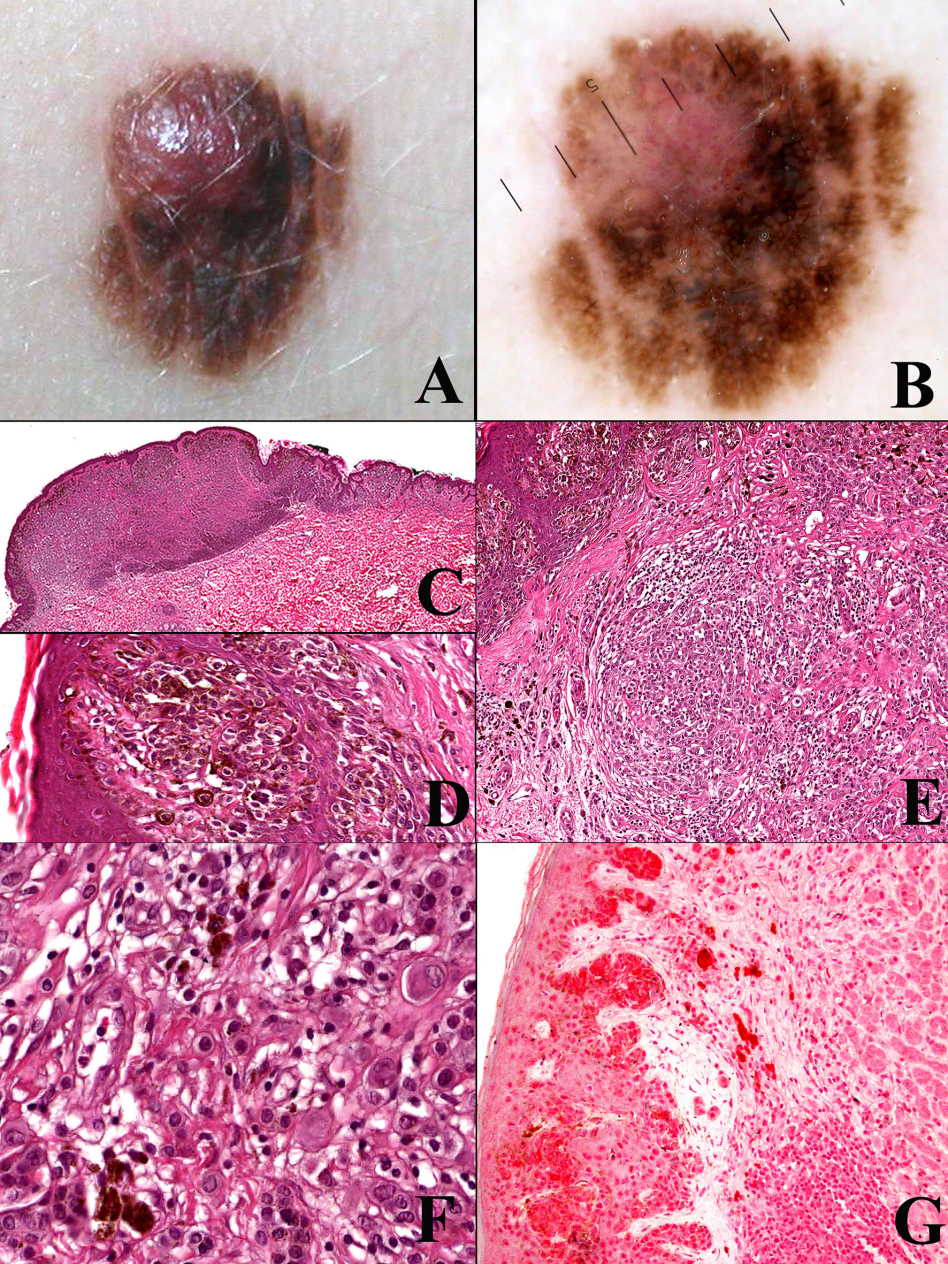
Man, 54 years; a severely atypical melanocytic tumor of the abdomen characterized by a flat pigmented area with an eccentric nodule **(A)**. On dermoscopy, the flat area is typified by a prominent and focally irregular pigment network, whereas the nodular area is characterized by an atypical vascular pattern **(B)**. Histopathologically, the tumor is strikingly asymmetric (**C**; hematoxylin–eosin, ×25), with a broad highly cellular “shoulder” composed by junctional melanocytes arranged in irregular nests and in single unit (**D**; hematoxylin–eosin, ×400); the severely atypical junctional component spans above the dermal nodule, the latter being characterized by a lymphoid cell infiltrate (**E**; hematoxylin–eosin, ×250) and nests of nevocytes intermingled with moderately pleomorphic epithelioid melanocytes with “inclusion-like” cytoplasms (**F**; hematoxylin–eosin, ×400); all the melanocytic components of this tumor were BRAFv600e mutated protein positive (not shown) and only the dermal epithelioid cell component disclosed loss of the nuclear expression of BAP1 (**G**; ×250). The tumor was interpreted as an early melanoma developing as a neoplastic progression of a common nevus and not as a progression of a BIN.

Many melanocytomas are instead dermal-based, thick, “combined” melanocytic tumors in which an activating mutation of *BRAF* (or, much less commonly, *NRAS*) is followed by a second genetic hit with expansion of a morphologically peculiar (“non-classical”) clone of melanocytes. Morphology of this secondary clone strictly depends on the type of second genetic hit: inactivation of the *BAP1* (*BRCA1*-associated protein) gene is the hallmark of *BAP1*-inactivated nevus (BIN) (33, 34); gain-of-function mutations of *CTNNB1* or loss of *APC* is found in deep penetrating nevus (DPN) (35, 36); loss-of-function of *PRKAR1A* is typical of pigmented epithelioid melanocytoma (PEM) (37, 38). However, several melanocytomas arise *de novo* (without a pre-exsisting common nevus): for example, cases of “pure” (non-combined) PEM are also genetically peculiar because often they harbor kinase (most commonly *PRKA*, but also *NTRK1* and *NTRK3*) (38) fusions as the initiating event. Most of these dermal-based tumors are clinically stable; however, they can display various degrees of histopathological atypia (39–42). Increasing atypical histopathological features may correlate with increased risk of disease progression (43), but available data are too weak because of the relative rarity of these tumors and the need of long-term follow-up data. Since the initiating genetic change of such neoplasms is often an activating mutation of *BRAF* or *NRAS*, the three above-mentioned types of melanocytomas are placed within Pathway 1 of melanomagenesis, whose endpoint is superficial spreading melanoma; however, cases of superficial spreading melanoma dysplaying the genetic signature of the above-listed melanocytomas are exceedingly rare. Therefore, in real life such melanocytomas are probably unrelated to the vast majority of classical (Clark’s and McGovern’s) (25, 26) types of melanoma. **Figure 1** shows a case of early superficial spreading melanoma over a combined BIN, with the malignant component being BAP1-positive, and being thus unrelated with the dermal melanocytoma.

According to Table 2.06 of the WHO classification (16), even the other pathways to melanoma starting from the respective nevi have their own “melanocytomas”, namely: atypical Spitz tumor (Pathway 4), (atypical proliferative) nodule in congenital nevus (Pathway 7), and (atypical) cellular blue nevus (Pathway 8). It has been suggested that these entities share with BIN, DPN, and PEM the existence of a “spectrum within the spectrum” (43), namely: a set of atypical histopathological features which can be variously combined with each other, thereby bearing a “spectrum” of lesions with increasing risk of disease progression up to overtly malignant neoplasms. However, the WHO Working Group underlines that regarding Pathway 7, there is no convincing evidence that *bona fide* proliferative nodules in congenital nevi evolve into melanoma (44); and that regarding Pathway 8, a histopathological diagnosis of malignancy is straightforward for melanoma arising in blue nevus (45). Instead, regarding atypical Spitz tumor, it is acknowledged that there is the need of a “risk stratification” (46), evidently because neoplasms belonging to the Spitz lineage distribute along a spectrum of increasing histopathological atypia, with their malignant end being Spitz melanoma (14, 15).

**Figure 2:**
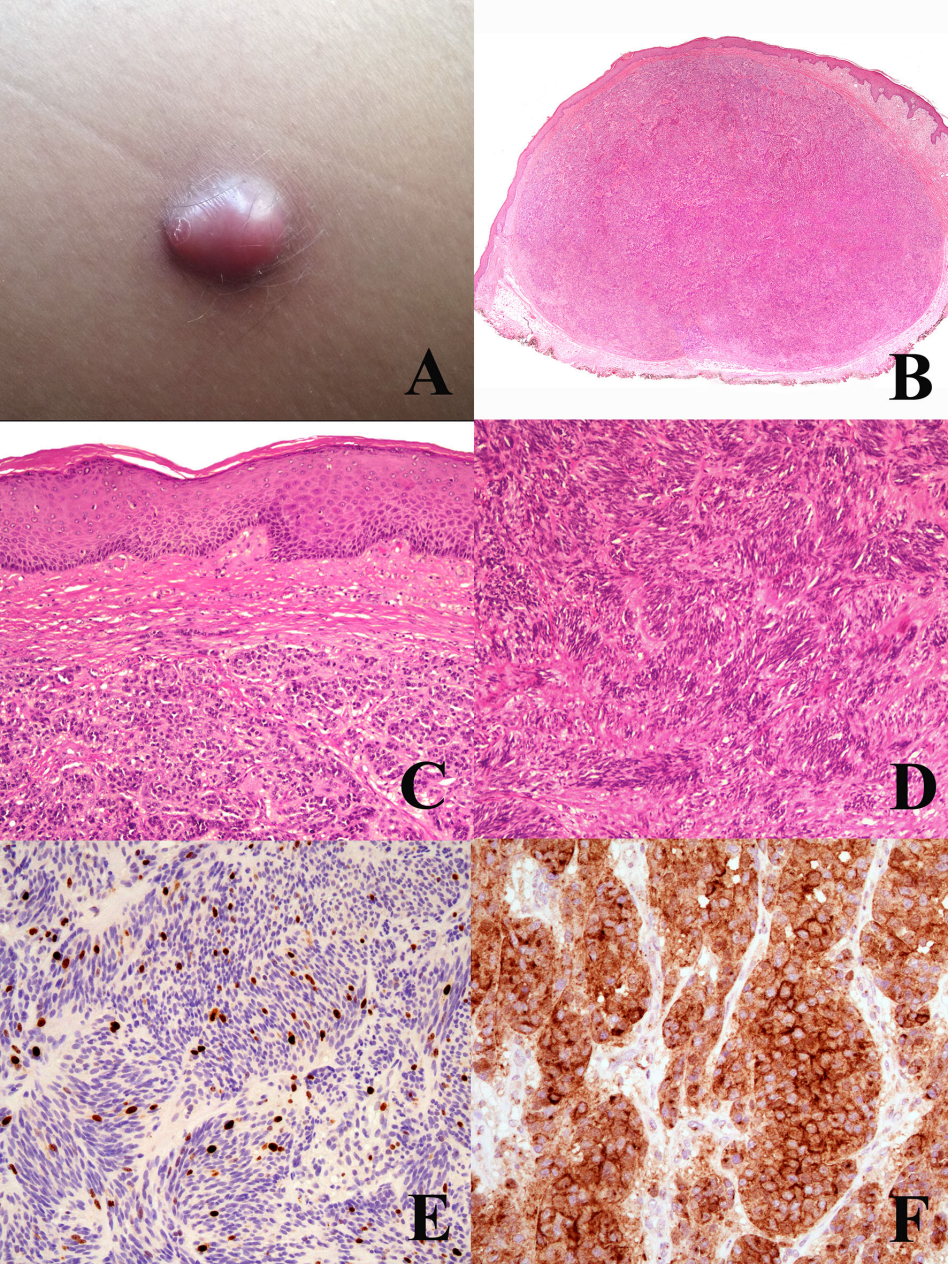
Woman, 44 years; a reddish nodule of the thigh **(A)**. Histopathology shows an expansile dermal nodule (**B** hematoxylin–eosin, ×25) composed by nests of epithelioid cells (**C** hematoxylin–eosin, ×250) and fascicles of spingle cells separated by thin fibrotic bands (**D** hematoxylin–eosin, ×250); the proliferation rate (Ki67-positive cells) is 5%, with no clusters of proliferating cells (**E**; ×250); the tumor cells are diffusely positive for TRKA (**F**; ×400). Molecular studies allowed to exclude the possibility of a dermal clear cell sarcoma and to establish a diagnosis of CRTC1-TRIM1 fused melanocytoma. Courtesy of Dr. Arnaud de la Fouchardière, Lyon, F.

Interestingly, atypical Spitz tumor shares at least with PEM a peculiar biological behavior, featuring a high incidence of nodal metastases with a very low incidence of distant metastases (41, 47): such as unique biological property that strongly favors ultrasonograpy monitoring over sentinel node biopsy in the clinical management of such cases (47, 48). Based on these data, PEM and atypical Spitz tumor might represent melanocytic tumors of low-grade (mostly lymphotropic) malignancy different from “classical” melanoma: it seems thus reasonable to include atypical Spitz tumor into the “melanocytoma” rubric, as suggested since the beginning (49). Interestingly enough, the list of putative low-grade melanocytic malignancies with a peculiar genetic and morphologic profile has been growing for the last years and has thus been increasingly supporting the concept itself (50–53). An example of CRTC1-TRIM11 (50) fused melanocytoma is provided in **Figure 2**; like several other melanocytomas, such a putatively low-grade malignant melanocytic tumor does not likely progress from a common nevus.

For the above, intermediate melanocytic tumors may be subclassified into: i) a “classical” subgroup (dysplastic nevus and melanoma *in situ*), which is placed within the morphologic and molecular progression spectrum of “classical” melanoma subtypes (superficial spreading and, possibly, nodular; WHO 2018 Pathway 1); and ii) a “non-classical” subgroup (“melanocytomas”) whose genetic pathways diverge from “classical” melanoma subtypes. Among the latter are probably low-grade melanocytic malignancies whose list has been increasing for the last years and whose risk stratification needs a careful and systematic approach (48).

Not surprisingly, neoplasms belonging to the WHO 2018 intermediate category are prone to a lower interobserver agreement and are classified as ambiguous by multiple pathologists. Thus, the intermediate rubric also encompasses the provisional categories IAMPUS, SAMPUS, and MELTUMP (6), whose definitions (see above) imply a “subjective” diagnostic uncertainty, rather than a morphologic subset of melanocytic neoplasms. Immunohistochemical and genetic investigations may help classify the WHO 2018 provisional entities into the proper subgroup of melanoytic tumors: this goal is of paramount importance because the “provisional” terminology should be adopted as less as possible (48).

## The WHO 2018 Progression Model: What Matters in Routine Practice

**Figure 3:**
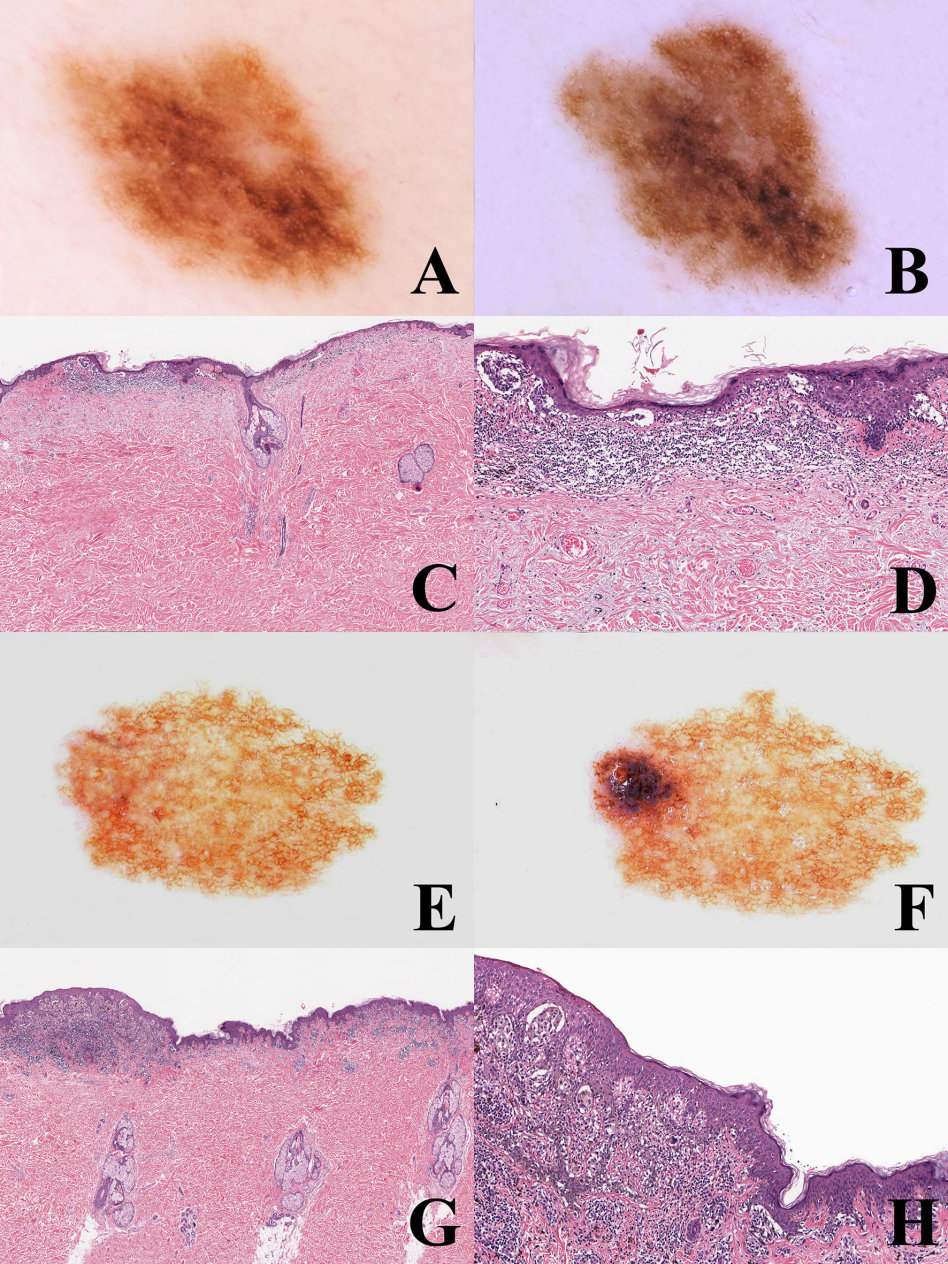
**(A–D)** man, 53 years; a pigmented lesion of the back with a slightly irregular pigment network **(A)**; after six months, the tumor appears as uniformly enlarged, with increasingly irregular pigment network **(B)**. Histopathologically, the tumor is strikingly asymmetric (**C**; hematoxylin–eosin, ×25), with a lichenoid infiltrate at the base of its more severely atypical half (**D**; hematoxylin–eosin, ×100). Even if the histopathological picture might be interpreted as a melanoma *in situ* developing in the background of a dysplastic nevus, the homogeneous remodeling of the tumor documented with dermoscopic digital monitoring favored the diagnosis of melanoma *de novo*. E-H: Woman, 35 years; a pigmented lesion of the back with a thin and regular pigment network at the baseline **(E)**; after eight months, a raised bluish areas is evident at the periphery (“dermoscopic island”) **(F)**. Histopathologically the tumor shares with the previous case the striking asymmetry **(G)** hematoxylin-eosin, ×25) and the presence of a lichenoid infiltrate at the base of its more severely atypical half **(H)** hematoxylin-eosin, ×100). However, dermoscopic digital follow up data clarify that this case likely represents an early melanoma *in situ* over a junctional dysplastic nevus.

**Figure 4:**
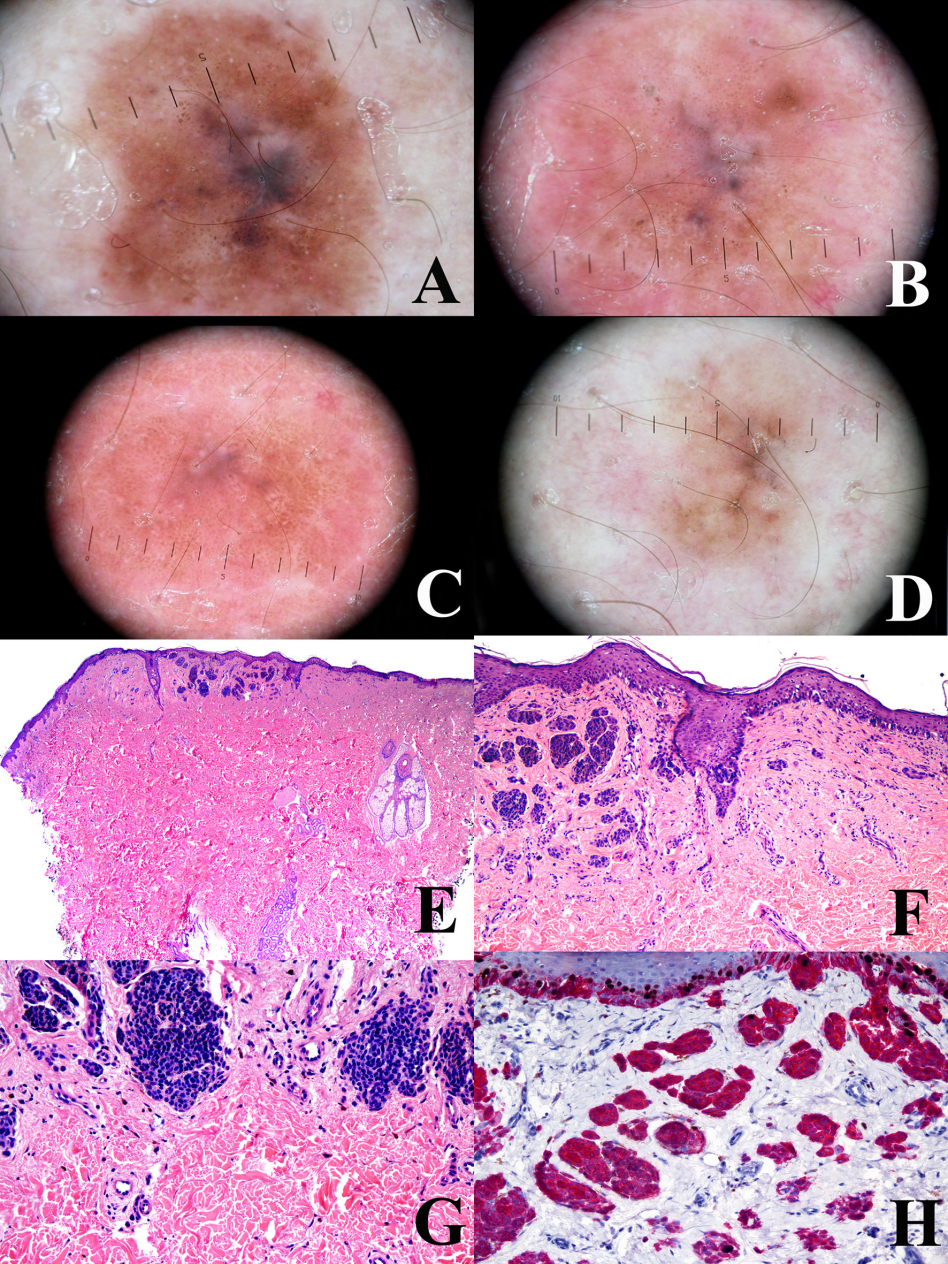
Man, 38 years at the time of the surgical excision of a pigmented lesion of the scapular area; at the baseline, the tumor shows a a relatively regular peripheral pigment network associated with slightly eccentric globules and a central bluish area **(A)** the tumor shows a progressive and relatively symmetric fading after 1 year **(B)**, four years **(C)**, and 6 years **(D)**. The tumor discloses a “trizonal” histopathological pattern (**E**; hematoxylin**–**eosin, ×25), with an atypical junctional component, a scar-like dermal thickening (**F**; hematoxylin**–**eosin, ×100) and a very bland-appearing deep dermal component (**G**; hematoxylin–eosin, ×100); the proliferation rate (Ki67-positive dermal melanocytes, evaluated with a KI67/MART1 double stain) is very low (**H**; ×250). These histopathological features are consistent with the so-called “sclerosing nevus with pseudomelanomatus features”. Such a histopathological diagnosis is in keeping with the slowly progressive and relatively symmetrical involution of the tumor, as documented with dermoscopic digital monitoring. Clinical images provided by Dr. Luigi Ligrone, Salerno, I.

The WHO 2018 progression model is aimed at giving a framework for a histopathological classification; it is therefore a relatively simplifed linear scheme which must be accepted with the awareness that not only are there multiple pathways to melanomagenesis but also that some of the intermediate steps may be bypassed and that other non-linear pathways exist. The most frequent and most important non-linear pattern is by far melanoma *de novo* of the “classical” type. In a meta-analysis carried out by Pampena et al. on 38 observational cohort and case–control studies, only 29.1% of melanomas likely arose from a preexisting nevus and 70.9% arose *de novo* (54). Studies on nevus-associated melanoma based on histopathology alone may have several biases: a benign component may be absent in the tissue levels examined or, else, it may be completely destroyed by the malignant growth; on the contrary, peripheral or deep areas of melanoma may have a deceptive “nevus-like” appearance (“pseudomaturation”). Dermoscopy and dermoscopic digital monitoring can help differentiate between melanoma characterized by a homogeneous remodeling of the tumor (likely melanoma *de novo;* **Figures 3A–D**) and melanoma characterized by focal changes (“dermoscopic island”; likely nevus-associated melanoma) (55) (**Figures 3E–H**). An early melanoma may be missed if grossing of the specimen is carried out blind to the clinicodermoscopic features of a given melanocytic lesion (56). Dermoscopic digital monitoring also shows that the overwhelming majority of nevi are stable and are more likely to involute according to one of the following: i) a fading pattern (progressive replacement of the nevus by normal skin); ii) a haloed pattern (progressive replacement of the nevus by centripetal extension of a peripheral white vitiligo-like ring); iii) a regression-like pattern (replacement of the nevus by dermoscopic regression structures (peppering, white scarlike ares) (57). The regression-like pattern is seldom documented with dermoscopic monitoring, but is peculiar enough to allow a clinicopathological differential diagnosis between melanoma with regression and its main benign simulator, the so-called “sclerosing nevus with pseudomelanomatous features” or “compound nevus with regression-like fibrosis” (58, 59). The latter is a kind of “chronically recurrent nevus” following chronic unnoticed trauma, and has been described mainly, albeit not exclusively, in the convex area of the back of young to middle aged patients. Histopathologically, this neoplasm is usually large and asymmetric with a typical “trizonal” pattern featuring: i) an irregular junctional component with irregular epidermal hyperplasia and areas of prevailing single cell proliferation; ii) a significant area of dermal sclerosis with architecturally atypical melanocytic nests; iii) a residual, bland-appearing nevus tissue (very often with congenital nevus-like features) around and deep into the cicatricial tissue (**Figure 4**). The presence of a clear-cut benign dermal component is the main clue to the diagnosis, because regressing melanoma is usually not associated with a nevus. Such a severely atypical melanocytic tumor, in our experience often cautiously diagnosed as MELTUMP, can be indeed diagnosed with confidence when considering the proper clinicopathological setting; together with the many nevi in special sites (nevi with site-related atypia), it is an example of histopathological atypia probably unrelated with a signficantly higher risk of progression toward melanoma. This entity also underlines the role of clinically identifiable “environmental modifiers” (trauma, epilation, acute sun exposure) which may increase the histopathological features of atypia in nevi (2, 34) presumably without any impact in melanomagenesis.

As also underlined by the WHO Working Group in a paper published shortly after the 2018 Classification, the risk of an individual nevus progressing to melanoma has been estimated to be in the order of one in 33,000 or less per year (60). Therefore, from a practical point of view, we can conclude that:

1. the vast majority of nevi are, at worse, clinicopathological simulators and not precursors to melanoma;
2. besides esthetic reasons, indication to their excision is solely related to the impossibility to rule out melanoma on clinical grounds alone;
3. with the possible (but not universally accepted) exception of medium (1.5–20 cm) and large/giant (>20 cm) congenital nevi, which carry a definite size-related melanoma risk [up to 15% (61)], by no means the excision of a nevus must be viewed as a tool of primary prevention (“prophylactic excision”).

These statements also apply to dysplastic nevus and dysplastic nevus syndrome. The WHO Working Group defines dysplastic nevus as a clinically atypical, histopathologically benign junctional or compound melanocytic tumor, >4 mm in breadth on fixed sections (>5 mm clinically), with architectural disorder plus cytological atypia (62). The former is typified by irregular (horizontally oriented, bridging adjacent rete, and/or varying in shape and size) and/or dyscohesive nests of intraepidermal melanocytes plus increased density of non-nested junctional melanocytes (*e.g.* more melanocytes than keratinocytes in an area ≥1 mm^2^); the latter is evaluated on the basis of the highest degree of cytological atypia present in more than a few melanocytes as low grade (nuclei ≤1.5× larger than basilar keratinocytes, with small or absent nucleoli and uniformly hyperchromatic or dispersed chromatin, and with “random” variation in size and shape) or high grade (nuclei ≥ larger than basilar keratinocytes, with prominent nucleoli and coarse or peripherally condensed chromatin, and with slightly confluent variation in size and shape) (62). It is stated that nevi with high-grade dysplasia and/or with additional genetic alterations such as TERT promoter mutation should be considered for complete excision (62); this implies that a nevus with high-grade displasia needs no re-excision if already excised with clear margins.

Some studies are reported in which the degree of dysplasia is related with an increased melanoma risk (63–66); however, with the sole exception of a retrosective review considering the personal history of melanoma (66), these studies were histopathologically based, *i.e*.: they did not take into account the clinical features of risk of the individual patients (familial history of melanoma, skin type, personal history of sunburns, number of nevi, number of clinically atypical nevi). Thus, from a practical point of view, a histopathological diagnosis of dysplastic nevus must be evaluated in the clinical context in order to assess the risk of the individual patient to develop a melanoma; and, since genetic findings are relatively inconsistent to date (62), the diagnosis of dysplastic nevus syndrome (aka: Familial Atypical Multiple Mole and Melanoma, FAMMM; OMIN #155600) is largely based on clinical criteria, *i.e.*: number of nevi, number of clinically atypical and/or large nevi, personal/famlial history of melanoma (64, 66).

**Figure 5:**
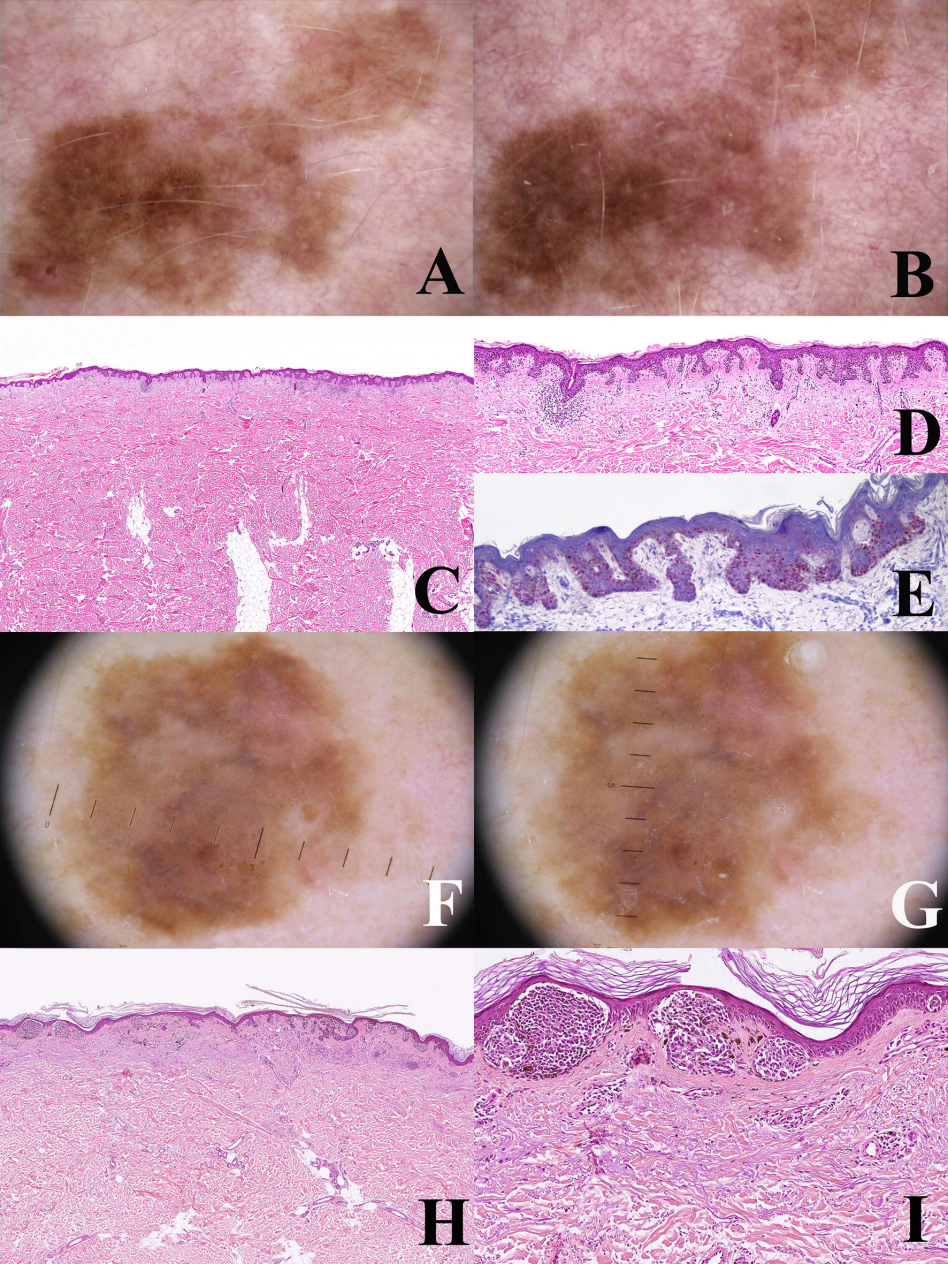
**(A–E)** Man 52 years. Dermoscopy of a large pigmented lesion of the back with an irregular pigment network at the baseline **(A)** after one year, the lesion shows an increase in size with a homogeneous remodeling and a more prominent pigment network **(B)** such a slow clinical evolution is akin to a lentigo maligna of chronically sun-exposed skin and virtually excludes a diagnosis of nevus. Histopathologically, the tumor has a dysplastic nevus-like silhouette (**C**; hematoxylin–eosin, ×25) but is severely atypical because of the striking predominance of tightly packed single melanocytes at the junction (**D**; hematoxylin–eosin, ×100). PRAME immunostain shows a strong and diffuse nuclear positivity in intraepidermal melanocytes **(E)** ×250), as expected in melanoma. Clinicopathological features of the lesion are diagnostic for lentiginous melanoma *in situ*. **(F–I)** Man, 59 years. A large pigmented lesion of the abdomen, dermoscopically characterized by tiny eccentically grouped globules and structureless peripheral areas **(F)** after seven months the peripheral strucureless areas show a clear-cut increase in size **(G)**. Histopathologically there are some areas with a dysplastic nevus-like silhouette, but the epidermis is largely atrophic (**H**; hematoxylin**–**eosin, ×25) and junctional nests are very large and irregular (**I**; hematoxylin–eosin, ×250). These features suggest a diagnosis of melanoma *in situ* with a focally “nested” architecture.

Excluded from the rubric of dysplastic nevus is lentiginous nevus, because being very common, unassociated with a relevant risk of progression to melanoma, and prone to poor diagnostic riproducibility (67). Lentiginous nevus is defined as a benign, junctional, or compound melanocytic tumor, <4 mm in width (on fixed sections), usually symmetrical but with poorly defined borders, with increased density of regularly spaced, non-nested junctional melanocytes around the tips and sides of the rete ridges, with no to mild cytological atypia and minor/variable features also seen in dysplastic nevi (67). These definitional features must be kept in mind because not uncommon in clinical practice are broad and irregular lentiginous melanocytic proliferations of the trunk and the proximal limbs, mostly found in elderly patients, which are probably the clinicopathological counterpart of lentigo maligna on non-chronically sun-exposed skin and are called lentiginous melanoma (68, 69). Dermoscopic digital monitoring of some of these lesions has demonstrated a homogeneous remodelling over many years, thereby suggesting that these are very slow-growing melanomas *de novo* and not the evolution to melanoma from lentiginous nevi (**Figures 5A–E**). In our experience on lentiginous melanoma, histopathological criteria alone are often weak and may result in a provisional diagnosis of IAMPUS or SAMPUS; the clinical picture of these cases is, however, very often unequivocal for melanoma and must be therefore incorporated into the decision-making process regarding their management.

Nested melanoma (of the elderly) is another example of deceptively bland melanoma (70) whose recognition often depends on a thorough clinicopathological correlation. Like lentiginous melanoma, it is often removed from the trunk and limbs in elderly patients as being large, growing and dermoscopically atypical flat pigmented tumor (71); histopathology features a junctional nesting which is not invariably irregular enough to allow a confident histopathological diagnosis; thus, the result is often a provisionla diagnosis of high-grade dysplasia, IAMPUS, or SAMPUS which, however, is not consistent with the clinical picture. Dermoscopic features of nested melanoma (70) suggest that it conceivably a slow growing melanoma *de novo*, rather than a melanoma evolving from a nevus (**Figures 5F–I**).

## A Management-Based Approach: The MPATH-Dx System and Beyond

A histopathological diagnosis is aimed at giving a Mutidisciplinary Team the main (albeit not the sole) information for the clinical management. However, such an approach centered on histopathology having some major limitations, more or less explicitly underlined by the WHO Working Group, namely:

1. the diagnostic terminology varies depending on the individual cultural background and on local giudelines (72);
2. the diagnostic interobserver reproducibility is poor even among experts (73);
3. all the available evidence-based clinical guidelines are set upon a dichotomic diagnostic approach (all melanocytic tumors are either nevi or melanomas) and upon a unifying concept of melanoma (all melanocytic malignancies have the same biological behavior which can be predicted on the basis of a universally applicable set of histopathological parameters) (3).

In 2014, the Melanocytic Pathology Assessment Tool and Hierarchy for Diagnosis (MPATH-Dx) schema was proposed in an effort to reduce uncertainty and offer guidelines, mostly for melanocytic tumors different from melanoma (the “classical” melanocytic malignancy with its own evidence-based guidelines) (74): notably, the original schema excluded some melanocytic tumors (pigmented spindle cell; Spitz; epithelioid blue; cellular blue; deep penetrating/plexiform spindle cell) from Class 1 (no apparent risk), thereby anticipating the WHO 2018 concept of intermediate melanocytic tumors. The MPAT-Dx system stratified melanocytomas into four classes (Classes 2 to 5) of melanocytic tumors, with the first two being discriminated on the basis of the degree of histopathological atypia, and the last two discriminated on the basis of Breslow’s thickness. The latter criterion, however, should not be applied to melanocytomas, because they are morphologically, genetically, and biologically different from “classical” melanoma with its “classical” prognostic parameters.

In order to specifically address the clinical management of dermal-based tumorigenic “intermediate” melanocytic tumors, practical recommendations have been delivered by the ESP, the EORTC, and the EURACAN (48). Morphological evaluation of these tumors is based on the evaluation of a list of general criteria, both architectural (diameter >6 mm; asymmetry; epidermal effacement; ulceration; high dermal cellularity; tumor clones; loss of grenz zone; absence of vertical “maturation”; expansile nodule formation; destriucive growth pattern; deep subcutaneous extension; pagetoid spread) and cytological (cellular pleomorphism; macro-eosinophilic nucleoli; variable density of nuclear chromatin; irregular nuclear membrane; >1 mitosis/mm^2^; overlapping nuclei; tumor necrosis). Melanocytomas are then stratified into “low-grade” (few criteria present) and “high grade” (roughly up to half of them present), with excision margins estimated as adequate at 2 mm for the former and at 5–10 mm for the latter. Since a 2-mm excision margin is recommended for every melanocytic tumor, no further excision is required for low-grade melanocytomas. Pigmented epithelioid melanocytoma is by definition an intermediate-high-grade tumor; sentinel node staging is recommended only for “unclassified atypical dermal tumors” and for cases in which a Spitz melanoma cannot be ruled out; cases labeled as MELTUMP should be managed as per melanoma of the same thickness.

**Figure 6:**
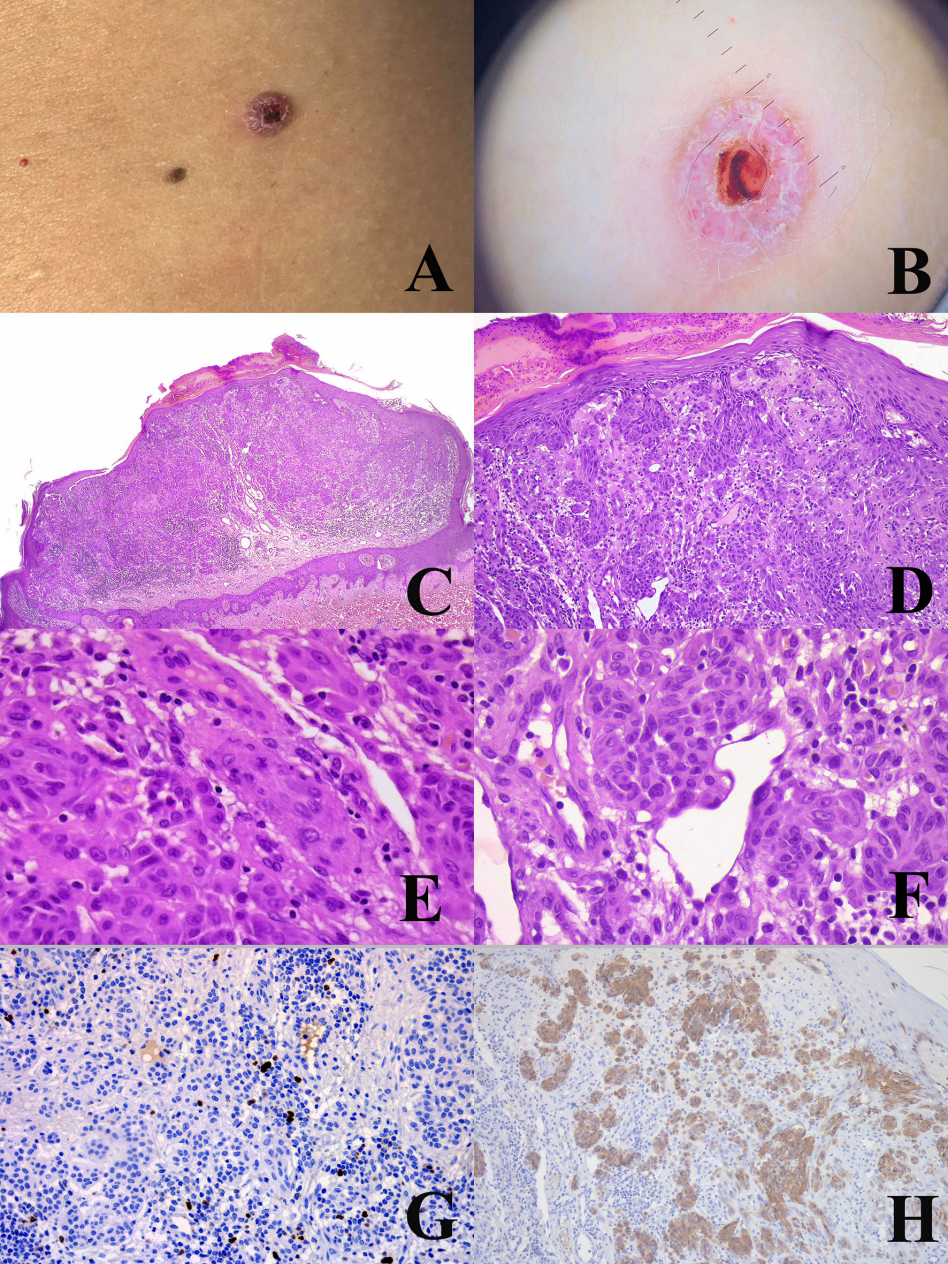
Woman, 22 years. An ulcerated nodule of the right flank **(A)** dermoscopically characterized by keratoacanthoma-like features with vessels surrounded by a white halo **(B)**. Histopathologically, the tumor has an irregularly nodular, exophytic silhouette with an epidermal “collarette”, a superficial crust, and a “brisk” inflammatory infiltrate in the dermis (**C**; hematoxylin–eosin, ×25); the superficial nests are very irregularly confluent with no sharp circumscription from the overlying epidermis (**D**; hematoxylin–eosin, ×250); dermal melanocytes show a “spitzoid” morphology, with spindle (**E**; hematoxylin–eosin, ×400) and epthelioid (**F**; hematoxylin–eosin, ×400) cells, both with reatively abundant and eosinophilic cytoplasms. In spite of the severe architectural atypia, the proliferation rate of the tumor (Ki67-positive dermal melanocytes) is low **(G)** ×250); however, the tumor is not an atypical Spitz tumor, but a classical nodular melanoma because it is positive to the antibody anti-BRAFv600e-mutated protein **(H)** ×250).

The ESP-EORTC-EURACAN recommendations concerning Spitz melanoma should be applied also on the basis of the recent observation that a “spitzoid” morphology is not invariably associated with a “Spitz” genetic signature (14, 15); in other words, malignant Spitz tumor (Spitz melanoma) is different from “spitzoid” melanoma, which can be regarded as a melanocytic malignancy with “Spitz-like” morphology but genetically ascribed to a “classical” melanoma subtype because of the presence of a specific driver mutation, or numerous DNA copy number changes, or a high TMB. **Figure 6** illustrates the clinicopathological features of an ulcerated melanocytic malignancy histopathologically composed of large epithelioid cells with Spitz-like features, but immunohstichemically typified as a “classical” melanoma because of its immunohistochemical positivity to the anti-BRAF mutated protein VE1 antibody. Parenthetically, PEM-like (75, 76) and DPN-like melanomas (77, 78) might be differentiated from their “melanocytoma counterpart” based on immunohistochemical and/or genetic findings akin to “classical” melanoma.

Based on the above, a new problem is thus rising in dermatopathology, *i.e.*: the differential diagnosis between severely atypical melanocytoma and melanocytoma-like “classical” melanoma. This is not merely a speculative problem, because both a severely atypical melanocytoma and a melanocytoma-like “classical” melanoma will likely spread to the regional nodes, but only the latter will be candidates to sentinel node biopsy and, possibly, to an adjuvant therapy with *BRAF*-inhibitors or with immune checkpoint inhibitors (79, 80). This means that underdiagnosing a “classical” melanoma as a severely atypical melanocytoma may address the patient to an improper wait-and-watch strategy. Many melanocytomas (comprising Spitz tumors) currently lack an identifiable genetic “signature”; by definition, however, they lack *BRAF*-mutation and a high TMB which are predictive parameters for neoadjuvant therapy (79, 80). Thus, the differential diagnosis between a severely atypical melanocytoma with no known genetic signature and a classical “melanocytoma-like” melanoma may be approached by looking for predictive (rather than diagnostic) paramenters; the same might apply for cases provisionally labeled as MELTUMP or as unclassified atypical dermal lesion (48).

## A Therapy-Oriented Diagnostic Approach

When dealing with an atypical melanocytic tumor of the skin, the first step can be the differential diagnosis between a “classical” type of melanocytic tumor and a “melanocytoma” (comprising Spitz tumor). Immunohistochemistry can assist such a differential diagnosis as follows:

- The anti BRAF-mutated protein VE1 antibody identifies the subset of melanocytic tumors of the “classical” type harboring the *BRAF^v600e^* mutation (or a “combined” melanocytoma) (48, 81);
- The immunostain for BAP1 can document loss of the consitutive nuclear immunoreactivity in BAP1-inactivated melanocytic tumors (33, 34);
- The anti PRAME immunostain can assist the differential diagnosis between benign and malignant “traditional” melanocytic tumors (82); in our experience, particularly for lentiginous neoplasms and for the differential diagnosis between congenital nevus and nevoid melanoma;
- The anti-ALK, anti-TRKA, anti-MET, anti-HRAS-WT, and anti-ROS1 antibodies identify the subset of melanocytic tumors of the Spitz lineage with the respective kinase gene changes (48, 83, 84);
- The anti-beta catenin immunostain identifies the aberrant nuclear positivity definitional for DPN and related tumors (36);
- Tha anti-R1alpha can document loss of constitutive nuclear immunoreactivity in PEM with inactivating mutation or epigenetic inactivation of *PRKAR1A* (85).

An immunohistochemical panel aimed at a risk stratification can encompass:

- p16, which may disclose uneven immunoreactivity or “clonal” loss as an atypical feature (2, 48);
- HMB45, which may be unevenly distributed, with loss of the “gradient” pattern seen in benign tumors (2);
- Cell cycle-related protein Ki67, which may show a high rate of expression and/or “proliferative clusters” in atypical lesions (2).

The traditional four-probe (targeting *MYB*, *RREB*, *Cep6*, and *CCND11*) plus the anti-*CDKN2A*/*Cep9* dual probe FISH examination may help refine the risk stratification of melanocytic tumors as recently proposed (86).

If morphology and immunohistochemistry are not contributory in assigning the melanocytic tumor to a given lineage, molecular analysis guided by morphology may be implemented as follows:

- Identification of hotspot mutations of *BRAF* (codon 600) and *NRAS* [exon 2 (odons 12, 13), exon 3 (codons 59, 61), and of exon 4 (codons 117, 146)];
- Sequencing techniques for the following: *NF1*, *KIT* (exons 11, 13, 17, and 18), *BRAF* (rare mutations), *NRAS* (rare mutations), and *MAP2K1* (exons 2 and 3; in-frame deletion) for “classical” melanocytic tumors; *GNAQ* (exons 4 and 5), *GNA11* (exons 4 and 5), *PLCB4*, and *CYSLTR2* for dendritic melanocytic tumors (WHO 2018 Pathways 8 and 9); *HRAS* (exons 2 and 3) for a subset of Sptz tumors; TERT promoter for a subset of aggressive malignencies (some characterized by a 'Spitz-like' morphology);
- Fluorescence *in situ* hybridization (FISH) or reverse transcriptase polymerase chain reaction (RT-PCR) examination for fusions involving: *BRAF* and *RET* for Spitz tumors; *MAP3K8* for morphologically malignant epithelioid cell Spitz neoplasms (87, 88); *PRKCA* for PEM.

As per ESP-EORTC-EURACAN guidelines, if the immunohistochemical screening implies additional procedures, immuno-positive cases (of Spitz neoplasms) should be confirmed for the respective genomic aberration by molecular examinations (48); this is, however, a theroretically uncommon scenario.

As a final step for an approach akin to tumor-agnostic therapy, NGS analysis can help identify melanocytic tumors with “rare” genetic signatures, and—even more important—melanocytic tumors with a high TMB which should be definitely ascribed to the category of classical melanoma with the relative therapeutic options. Specialized referral centers must be involved for sequencing, fusion studies, and NGS examination (48).

**Figure 7:**
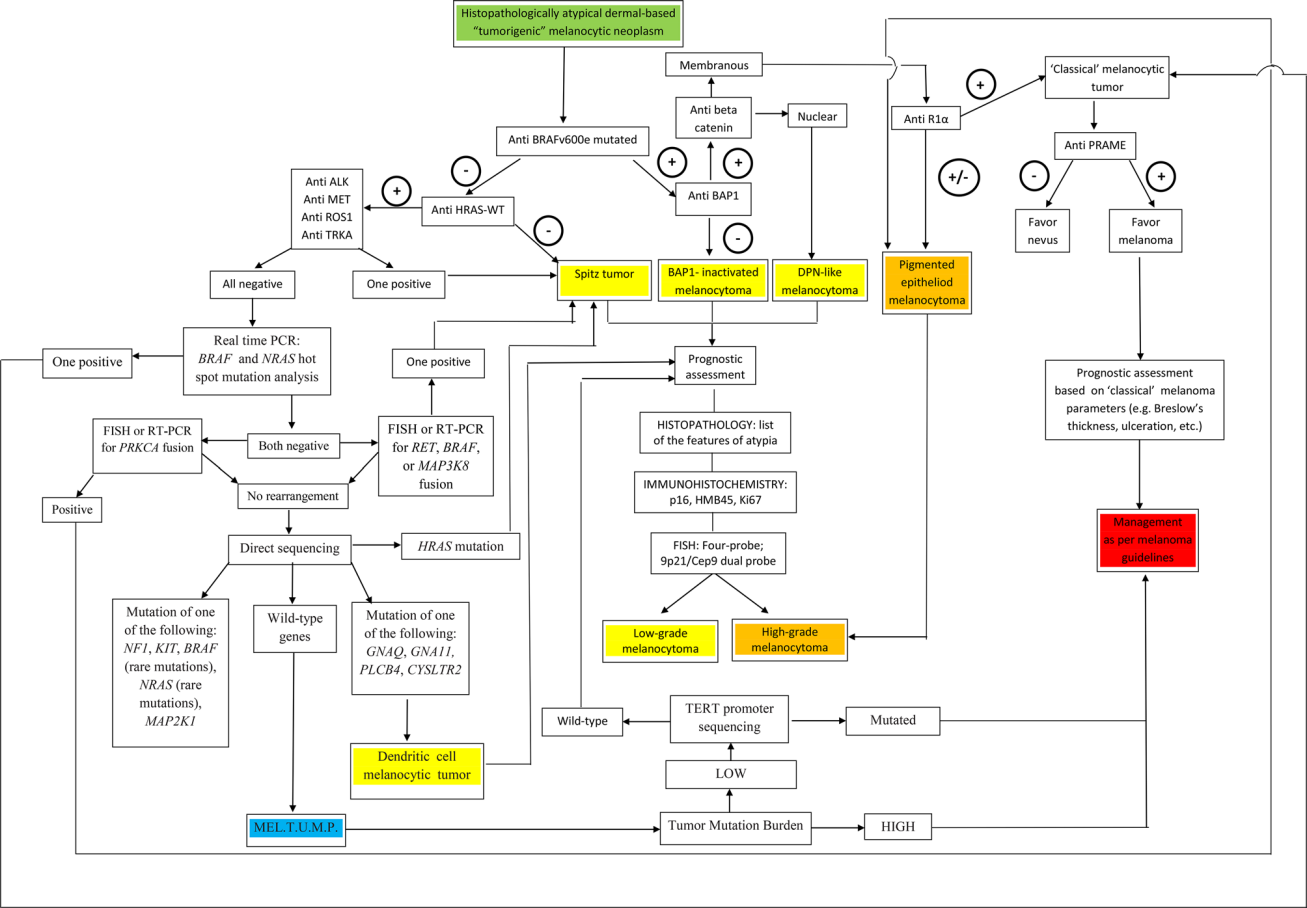
A flow chart illustrating a therapy-oriented morphomolecular approach to atypical dermal-based tumorigenic melanocytic neoplasms. Of paramount importance are: i) the distinction between melanocytomas (recognized as such by specific genetic signatures) and melanocytic tumors of uncertain malignant potential (MEL.T.U.M.P.; provisionally defined as tumors with unknown driver mutations); ii) among melanocytomas, the distinction between low-grade and high-grade tumors; iii) among MELTUMP, the distinction between tumors with a low tumor mutation burden and tumors with a a high tumor mutation burden, the latter being best managed as per “classical” melanoma.

A visual summary of the above-proposed algorithmic diagnostic approach is given in **Figure 7**.

## Take-Home Message

The traditional “dichotomic” (benign *vs* malignant) view of melanocytic tumors and the concept of melanoma as a “unique” clinicopathological entity no longer fit with the routine diagnostic approach. Along with “classical” (Clark’s and McGovern’s) subtypes of melanoma, other melanocytic malignancies, each charcaterized by peculiar biological behavior probably exist, must be distinguished from “classical” melanoma subypes and require specific clinical guidelines. Clinicopathological correlation can allow both reducing the histopathological diagnostic uncertainty and addressing patients to a proper management.

## Author Contributions

Both authors listed have made a substantial, direct, and intellectual contribution to the work and approved it for publication.

## Conflict of Interest

The authors declare that the research was conducted in the absence of any commercial or financial relationships that could be construed as a potential conflict of interest.
